# A mini-review: photodynamic therapy-induced immune activation

**DOI:** 10.3389/fphar.2026.1746961

**Published:** 2026-01-21

**Authors:** Xiaowen Cai, Albert Wing Nang Leung, Le Lv, Chuanshan Xu

**Affiliations:** 1 School of Applied Biology, Shenzhen City Polytechnic, Shenzhen, China; 2 School of Applied Biology, Shenzhen Institute of Technology, Shenzhen, China; 3 School of Graduate Studies, Lingnan University, Tuen Mun, China; 4 Guangzhou Municipal and Guangdong Provincial Key Laboratory of Molecular Target & Clinical Pharmacology, The NMPA and State Key Laboratory of Respiratory Disease, School of Pharmaceutical Sciences, Guangzhou Medical University, Guangzhou, China

**Keywords:** immunotherapy, mechanism, photodynamic therapy, photosensitiser, tumor

## Abstract

Tumors are considered to be among the most significant threats to human health. Immunotherapy, which is achieved through the body’s own immune response, shows great potential in the treatment of tumors. Nevertheless, the current low response rate in practical applications still needs to be overcome. Photodynamic therapy (PDT) is a minimally invasive treatment method that generates reactive oxygen species (ROS) through light irradiation of photosensitizers (PSs). It has been demonstrated that PDT is capable of not only efficiently eradicating tumors, but also effectively activating the immune system to recognize and destroy them. In addition, it has been demonstrated that the activation exhibits a persistent anti-tumor effect. It is evident that PDT demonstrates significant potential in the treatment of tumors, the inhibition of metastasis and the prevention of recurrence. This review summarizes the specific mechanisms of PDT-induced immune activation, including innate immunity and adaptive immunity, lists the relevant applications of organic and inorganic PSs in this field, and discusses the next challenges for PDT in tumor immunotherapy.

## 1 Introduction

Immunity can be defined as the body’s natural defense mechanism against foreign pathogens (Shen et al., 2025). In the presence of foreign substances within the body, macrophages are able to recognize non-self components and release signal molecules to activate effector cells (Joseph et al., 2025). This process ultimately results in the elimination of foreign substances and the induction of a therapeutic effect. The utilization of the immune system of the body to treat diseases has already shown unique advantages in various fields (Zhang et al., 2025; Yue et al., 2025; Akhtar et al., 2024; Chen S. et al., 2024; Bartosińska et al., 2025; Domka et al., 2023; Domka et al., 2024; Patil et al., 2023). In the domain of oncology, immunotherapy can reduce the occurrence of adverse effects when compared with alternative therapeutic modalities (Barjij and Meliani, 2025). Immunotherapy has also been identified as a leading treatment modality in the 21st century, with significant advancements being demonstrated in recent years. However, the prevailing single immunotherapy frequently exhibits inadequate therapeutic intensity, thus hindering its capacity to achieve complete disease eradication (Kah and Abrahamse, 2025). For instance, in the application of immune checkpoint blockade, a major branch of immunotherapy, the clinical non-response rate is relatively high due to individual differences (Aebisher et al., 2024a). Consequently, the exploration of novel solutions to bolster tumor immune responses may provide a renewed prospect for the efficacy of this therapeutic approach.

**FIGURE 1:**
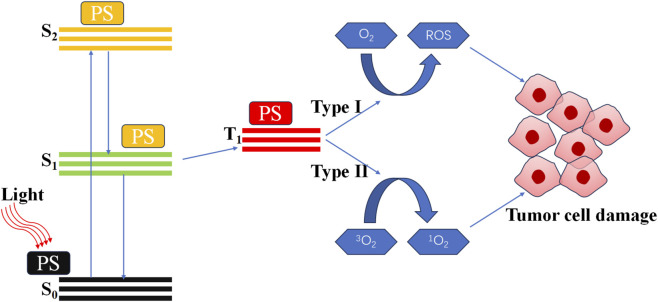
Mechanism and application of photodynamic therapy.

*In vitro*, stable and controllable non-destructive physical stimulation represents a highly effective method for activating the immune system (Adkins et al., 2014). These physical factors include light, sound, X-rays and other forms of electromagnetic radiation. Among these, photodynamic therapy (PDT) has been identified as a highly effective solution to enhance immunotherapy (Hwang et al., 2018; Garg et al., 2010). PDT is a treatment modality that employs light activates photosensitizer (PS) to generate reactive oxygen species (ROS) (Gore et al., 2025; Yang Y.-C. et al., 2023). The specific mechanism of PDT is illustrated in Figure 1 (Alvarez and Sevilla, 2024; Austin et al., 2025; Cai et al., 2025; Dobson et al., 2018). It was widely accepted that the therapeutic effect of PDT was a direct consequence of ROS. Excessive ROS can induce damage to DNA and proteins, thereby directly killing cancer cells (Wei et al., 2024; Agostinis et al., 2011). This constitutes the primary and most effective mechanism of PDT. And in the context of recurrent tumors and drug-resistant infections, PDT-induced immunotherapy has been shown to have significant potential (Xu et al., 2023; Gunaydin et al., 2021; He et al., 2023).

## 2 Photodynamic immunotherapy on tumor

Following the observation of tumor regression in cases of acute bacterial infection by William Bradley Coley, research into utilizing the body’s immune system to treat tumors has undergone significant expansion (Tsung and Norton, 2006). This approach is regarded as a non-invasive and effective means of preventing and treating tumors. However, during the application, it has been found that the differences in individual immune environments can lead to varying therapeutic effects. In certain instances, these disparities may even give rise to immunotherapy-resistant cases, thereby significantly hindering the advancement of immunotherapy. In order to enhance the immune response rate, researchers have initiated the exploration of combinations of immune and other alternative therapies. In certain studies, the combination of physical therapy, chemotherapy, gene therapy and immunotherapy has demonstrated synergistic effects and complementary advantages (Yang J.-K. et al., 2024). Specifically, the efficacy of physiotherapy and chemotherapy in reducing tumor volume, stimulating immunogenicity, promoting the release of inflammatory cytokines and antigens, and recruiting immune effector cells has been demonstrated (Kang et al., 2023; Huis et al., 2023). Among the aforementioned techniques, PDT is a non-invasive and controllable exogenous light stimulation technique that uses PS to produce ROS, thereby rapidly killing tumors (Cheng et al., 2022). In addition, PDT can stimulate immunomodulatory effects, which may enhance anti-tumor immunity and reduce the likelihood of metastasis and recurrence (Aebisher et al., 2024b).

Growing evidence shows that PDT has the capacity to activate both innate and adaptive immunity (Fang et al., 2025; Mazur et al., 2023; Rajan et al., 2024; Wahnou et al., 2023). An effective immune response can boost the elimination of tumors and the prevention of recurrence by maximizing local inflammatory responses and activating immune cells to destroy tumor tissues (Hua et al., 2021; Sorrin et al., 2020). Guided by the principles of cancer immunotherapy, an optimal PDT regimen should both destroy the primary tumor and induce immunogenic cell death (ICD). This activates the immune system to recognize and eliminate residual tumor cells, including distant metastases.

### 2.1 Immune activation induced by PDT

The anti-tumor effects of PDT are primarily attributable to three distinct mechanisms: ROS-induced cell death, damage to the tumor-associated vascular system and the initiation of tumor immune responses (Li M. et al., 2022). In this context, the immunogenicity of dead cancer cells has recently been identified as a pivotal factor in determining the efficacy of cancer therapy. Among these, ICD is a paradigm of highly effective cancer treatment, which triggers the activation of immune responses, resulting in strong and durable anticancer immunity specific for host cancer cells (Singh et al., 2024). A key feature of ICD is the emission of damage-associated molecular patterns (DAMPs). The ICD process involves the surface exposure and release of these molecules from dying cells. These released molecules serve as potent adjuvants, activating antigen-presenting cells. In turn, these cells phagocytose tumor debris, facilitate cross-presentation of antigens via major histocompatibility complex class I (MHC I) to CD^8+^ T cells, and thereby initiate a cellular immune response (Turubanova et al., 2019).

PDT has been demonstrated to be a strong inducer of innate immunity, which orchestrates significant anti-tumor effects. This innate activation is imperative for subsequently engaging the adaptive immune system (Jung et al., 2021). Key mechanisms include DAMP release by dendritic cells (DCs) to promote cross-presentation of tumor antigens to T lymphocytes (T cells), culminating in specific cytotoxic responses (Ding et al., 2025). Additionally, the resulting T cells can differentiate into memory cells, establishing long-term immunological protection against recurrence.

#### 2.1.1 Activation of innate immunity

**FIGURE 2:**
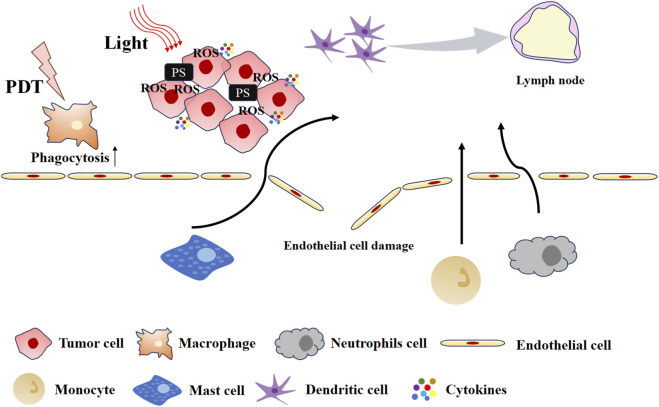
The inflammatory response induced by PDT.

It is considered that PDT-induced acute inflammation is the initial step in PDT-enhanced anti-tumor immunity (Henderson et al., 2004). The inflammatory response induced by PDT is illustrated in Figure 2. The inflammatory response is accompanied by the release of inflammatory cytokines and chemokines, including interleukin (IL-6 and IL-8), tumor necrosis factor (TNF), and interferon (IFN), among others (Brackett and Gollnick, 2020; Vanden Berghe et al., 2006). Collectively, these factors promote the influx of innate immune cells into tumors, where they attack tumor cells. The upregulation of acute-phase proteins (APPs) in serum promotes the maturation and release of neutrophil progenitor cells from the bone marrow (Mušković et al., 2023). PDT-induced photodamage to tumor vasculature and complement system activation jointly drive neutrophil migration to and infiltration of the tumor site (Fingar, 1996). Following PDT, neutrophils are also observed in tumor-draining lymph nodes (TDLNs), having entered through high endothelial venules (Kousis et al., 2007). Neutrophils exert anti-tumor effects both locally and systemically via two key mechanisms: direct destruction of tumor tissue and promotion of anti-tumor CD^8+^ T cell activation. In parallel, PDT also triggers other innate immune responses. For example, PDT induces high expression of HSP70 in tumor cells and activates the complement system, which together contribute to macrophage activation (Merchant and Korbelik, 2011). Additionally, the complement system is also activated to facilitate the dissolution of tumor cells.

#### 2.1.2 Activation of adaptive immunity

**FIGURE 3:**
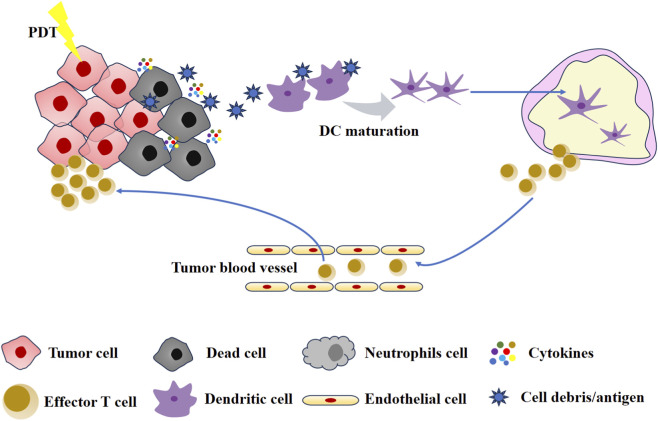
The adaptive immunity induced by PDT.

The adaptive immunity of PDT is attributable to ICD. Indeed, studies have demonstrated that PSs function as effective inducers of ICD (Du et al., 2025; Liu et al., 2024; Li Z. et al., 2022; Lee et al., 2025). The process stimulates the exposure of a considerable number of DAMPs. These DAMPs include calreticulin (CRT), high mobility group protein 1 (HMGB1), and adenosine triphosphate (ATP) (Barjij and Meliani, 2025). Subsequent to the release, the CRT migrates to the outer surface of the plasma membrane of dead tumor cells and is recognized by low-density lipoprotein receptor-related protein 1 (LRP1) receptors on DCs (Garg et al., 2012). The outcome of this process is the maturation of DCs and the completion of the antigen presentation process. Following activation, DCs migrate to the lymph nodes, where they in turn activate T cells and B cells, thereby triggering an acquired immune response. Furthermore, it has been observed that extracellular ATP binds to purinergic receptors P2Y2 (P2Y2R) and P2X7 (P2X7R) on DCs (Agostinis et al., 2011). This leads to the recruitment of DCs, thereby promoting the formation of inflammasome and the secretion of inflammatory stimulators. Finally, activated DCs and T cells mediate patient-specific immune effects that result in the elimination of both primary and metastatic tumors (Mroz et al., 2011). The adaptive immunity induced by PDT is shown in Figure 3.

By stimulating a systemic immune response to eradicate tumors, PDT functions as an immunogenic modality (Relvas et al., 2023). This action, analogous to administering an *in-situ* vaccine, underlies the synergistic potential of combining PDT with immunotherapy to robustly amplify the host’s antitumor immunity. However, given the numerous factors that influence PDT, current combination therapy methods primarily concentrate on enhancing the efficiency of PDT. In accordance with the three elements of PDT including “light, PS and oxygen” the corresponding strategies are designed through regulating light fluence, PS dose and location, and improving molecular oxygen in the tissues. For example, PDT with high doses of PS and light energy usually induces “accidental” necrosis (Cramer et al., 2022; Kessel, 2020). Necrotic cells release injury-associated molecular patterns, and promote mature DCs and cytotoxic T cells (Donohoe et al., 2019). The impact of apoptosis on the immune system is characterized by a “silent” or “anti-inflammatory” response. It is only through the immunogenicity-induced process of apoptosis that immune activation is triggered (Garg and Agostinis, 2014).

Notably, tumor hypoxia compromises PDT efficacy by driving immunosuppression: it upregulates chemotactic cytokines (CCL22 and CCL28), recruits immunosuppressive cells like myeloid-derived suppressor cells (MDSCs) and regulatory T cells (Tregs), and reprograms macrophages and neutrophils toward tumor-promoting phenotypes, collectively fostering a pro-tumor microenvironment (Yang et al., 2021; Corzo et al., 2010). Critically, hypoxia also directly impairs the effector functions of T cells and natural killer (NK) cells. Therefore, strategies to relieve tumor hypoxia are pivotal for enhancing PDT and its associated immunotherapy. A promising approach involves engineering nanomaterials capable of self-supplying oxygen. For example, Zhou et al. utilized catalase-loaded silica materials to decompose tumor H_2_O_2_ to produce oxygen, thereby alleviating hypoxia and eliciting a robust immune response characterized by M1 macrophage polarization, DC maturation, and CD^8+^ T cell infiltration (Xu et al., 2020; Liu et al., 2021; Zhou et al., 2025). This demonstrates the potential of combining PDT with oxygen-generating agents to induce potent antitumor immunity. Beyond enzyme-based systems, other inorganic nanomaterials with catalase-like activity, such as those composed of cerium (Ce), manganese (Mn), and platinum (Pt), have also been explored for *in situ* oxygen generation (Chen et al., 2023; Chudal et al., 2019; Wei et al., 2022). Furthermore, biohybrid strategies utilizing microorganisms have emerged. Certain microbes (e.g., Chlorella) harness photosynthesis within their thylakoid membranes, offering a novel biological method to elevate intratumoral O_2_ levels and potentiate PDT-mediated immunotherapy (Wang et al., 2021).

The immune efficacy of PDT is largely determined by the intracellular localization of the PS, which governs the mechanism of cell death and subsequent immune activation (Ji et al., 2022). Mitochondria-targeted PSs intensify mitochondrial oxidative stress, amplifying PDT-induced damage to promote antitumor immunity (Desai et al., 2024). Hydrophilic PSs often accumulate initially in lysosomes/endosomes, organelles integral to the autophagic and apoptotic death pathways engaged by PDT (Reiners et al., 2014; Plekhova et al., 2022). By contrast, the accumulation of amphiphilic or hydrophobic PSs in the endoplasmic reticulum (ER) markedly enhances therapeutic and immunogenic outcomes. This is because ER-localized ROS generation induces ER stress to stimulate the release of DAMPs including CRT, HMGB1, and heat shock proteins. Thus ROS production within the ER serves as a promising development strategy for effective ICD.

### 2.2 PSs in tumor photodynamic immunotherapy

The efficiency of PDT is determined by three fundamental factors: light, PS and oxygen. The PS functions as an essential intermediate link between the other two elements, playing a pivotal role in the overall process (Zhang et al., 2022; Chen M. et al., 2024). It is important to note that not all PDT regimens are capable of producing immune effects, thus the selection of an appropriate PS constitutes the most critical step in PDT-assisted immunotherapy for tumor (Turubanova et al., 2021). Table 1 presents a selection of instances illustrating the application of PS for tumor immunotherapy.

**TABLE 1:** Application of PSs for tumor immunotherapy.

Category	PS	Parameters	Ref
Inorganic	Cu_2_MoS_4_/Au Heterostructures	850 nm, 0.75 W/cm^2^ *in vitro*, 0.5 W/cm^2^ *in vivo*	Chang et al. (2020)
Inorganic	Au/Ag nanorod	1,064 nm, 1 W/cm^2^	Jin et al. (2021)
Inorganic	Ag^+^-Coupled Black Phosphorus Vesicles	660 nm, 150 mW/cm^2^	Li et al. (2020)
Inorganic	Black Phosphorus quantum dots Vesicles	680 nm, 150 mW/cm^2^	Li et al. (2019)
Inorganic	Au_44_ Nanoclusters	808 nm, 1.5 W/cm^2^	Yang et al. (2023b)
Inorganic	Au nanocages	808 nm, 1 W/cm^2^	Yang et al. (2022)
Organic	Aloe Emodin	450 nm, 0.1 W/cm^2^	Yu et al. (2025)
Organic	Hypericin	600–720 nm, 1.5 W/cm^2^	Kaleta-Richter et al. (2020)
Organic	Pheophorbide A	660 nm, 20 mW/cm^2^ *in vitro*, 5 mW/cm^2^ *in vivo*	Li et al. (2025)
Organic	Boron-dipyrromethene	808 nm, 1.5 W/cm^2^	Yang et al. (2024b)
Organic	Copper chlorophyllin-based carbon dots	405 nm, 10 mW/cm^2^	Kirbas Cilingir et al. (2024)
Organic	Hypocrellin B	645 nm, 0.8 W/cm^2^	Zhang et al. (2021)

#### 2.2.1 Inorganic PSs

Inorganic PS primarily encompasses chalcogenides, black phosphorus, graphite and metal nanoparticles, which characteristically exhibit elevated light conversion efficiency and ROS production, thereby evoking considerable interest (Guo et al., 2023). It is evident that Au nanoparticles have become ideal candidates for PSs, owing to their strong surface plasmon resonance effect. Jin et al. designed an Au/Ag nanorod with the capability of adjusting its surface plasmon resonance band to the near-infrared II window (Jin et al., 2021). This adjustment is made possible through the precise control of the thickness of the Ag shell, thereby allowing the Au/Ag nanorod to elicit both PDT and photothermal effects when exposed to near-infrared II light. As demonstrated in the experimental section, the study revealed that exposure of Au/Ag nanorods to 1,064 nm irradiation resulted in the induction of ICD in tumor site. In addition, it has been demonstrated that this process facilitates the maturation of DCs and the secretion of pro-inflammatory cytokines. Moreover, the process can induce an immune response in the body, thereby converting “cold” tumor into “hot” tumor and effectively inhibiting the growth of distant tumors in mice. This outcome is achieved through the combination of the Au/Ag nanorod with immune checkpoint inhibitors, thereby confirming that the treatment regimen triggered a strong immune memory effect.

In practical applications, some inorganic PS are difficult to degrade *in vivo*, thus research and development of biodegradable inorganic PS has attracted significant attention. Black phosphorus is an allotrope of phosphorus that can be prepared from white phosphorus or red phosphorus under conditions of high temperature and high pressure. The nanomaterials prepared from black phosphorus have adjustable band gaps and strong light absorption, thus being able to act as PSs or photothermal agents in PDT or PTT (Dong et al., 2022). Furthermore, the lone pair electrons on each phosphorus atom render black phosphorus more reactive to water and oxygen, and it is more readily biodegradable into non-toxic phosphate esters and phosphonic acid esters, making it safer. Li’s team initially constructed an Ag^+^ coupled black phosphorus vesicle (Li et al., 2020). In this combination, the strong charge coupling of Ag^+^ enhances the absorption of black phosphorus quantum dots in the near-infrared II region and is accompanied by a strong photoacoustic signal. This technology can be employed to facilitate accurate and in-depth biological tissue imaging, thereby providing a framework for guiding and monitoring the subsequent tumor treatment process. In tumor-bearing mouse models, the treatment with vesicles and 660 nm laser irradiation not only inhibited tumor growth in mice but also recruited immune cells for tumor immune responses. The enhanced immune effect of PDT was also verified in a mouse lung metastasis model.

Inorganic PSs are valued for their potent photodynamic properties and photostability, yet their inherent low biodegradability poses a significant barrier to clinical use. Persistent accumulation in tissues raises concerns about potential long-term toxicity (De Matteis, 2017). This has spurred the development of biodegradable or biocompatible material platforms as a clinically transformative direction. Furthermore, the biological fate and safety of these materials are profoundly influenced by their physicochemical properties, such as surface charge. Positively charged nanoparticles, for example, show enhanced mucosal interaction coupled with reduced retention, a profile that may improve safety (Wang D. et al., 2023). Consequently, achieving optimal biocompatibility through controlled degradation and tailored surface design is a paramount objective for the clinical development of next-generation inorganic PSs.

#### 2.2.2 Organic PSs

The application of organic PSs is more extensive (Jia et al., 2023). As early as 2004, the first-generation PS porphyrin was reported to be capable of inducing ICD in mouse colon cancer cells through PDT and initiating the immune response in mice (Jalili et al., 2004). Subsequent to that, the field of PDT-induced tumor immunotherapy has undergone rapid development (Lu et al., 2023; Nasr Esfahani et al., 2024; Chen et al., 2025; Zhao X. et al., 2024). For instance, Yu’s team demonstrated a sophisticated understanding of the coordination ability of aloe-emodin with multivalent metal ions, specifically copper, to construct targeted nanoparticles (Yu et al., 2025). These nanoparticles were loaded with copper ions and aloe-emodin, thus offering a multifaceted approach to targeted drug delivery. The efficacy of these nanoparticles in activating antigen-presenting cells in tumor-bearing mice under 450 nm light excitation has been demonstrated. In addition, they have been demonstrated to activate T cells, promote the transformation of CD^8+^ T cells into central memory T cells, inhibit the activation of MDSCs, and promote the transformation of macrophages into M1. The induction of a strong adaptive immune response is of considerable therapeutic significance.

5-Aminolevulinic acid (5-ALA) is a naturally occurring amino acid that has the capacity to synthesise protoporphyrin IX (PpIX) via the heme biosynthesis pathway (Wang L. et al., 2023). It has been established that heme biosynthesis is more active in tumor cells than in normal cells. Consequently, PpIX accumulates more in tumor cells, thus providing a natural advantage for the anti-tumor treatment of 5-ALA. In Zhao’s study, it was established that exosome release following 5-ALA treatment of skin squamous cell carcinoma, despite the absence of any cytotoxic effect on tumor cells, could induce the maturation of DCs and the secretion of IL-12, thereby enhancing anti-tumor immunity (Zhao et al., 2020).

In contrast to inorganic PSs, organic PSs demonstrate enhanced biocompatibility and synthetic tunability, key factors underpinning their clinical adoption (Lu et al., 2023; Chen et al., 2025). Notably, most PSs currently used in the clinic are small organic molecules or specific transition metal complexes, a preference attributable to their desirable pharmacokinetic and biophysical characteristics (Zhao D. et al., 2024). The field, however, faces enduring challenges, primarily the shallow penetration depth of light at typical PS excitation wavelengths (400–700 nm) and the hypoxic nature of tumors.

In response, there is a marked shift toward exploring natural products as novel PSs (Aziz et al., 2023). These agents offer the combined benefit of eliciting ICD via PDT and directly modulating the tumor immune microenvironment. A prominent case in point is hypericin, a naturally occurring ER-localizing PS. Upon light activation, it produces a sharp increase in ROS within the ER, culminating in significant ER stress and the induction of potent, immune-mediated antitumor effects (Xu et al., 2025). Complementing this, hypericin has been shown to decrease pro-tumor M2 macrophage populations, retard tumor growth, and boost infiltration of cytotoxic T cells into the tumor core (Buľková et al., 2023). Thereby, it helps establish a proinflammatory niche essential for activating adaptive immunity. The distinctive value of natural PSs thus lies in this dual functionality: competence in triggering ICD and an inherent capacity to remodel the immunosuppressive microenvironment, positioning them as promising and distinctive agents for the future of antitumor immunotherapy.

#### 2.2.3 Advanced PSs

In addition to the above-mentioned PS, new types of PS are also being developed to cope with the complex microenvironment of tumor immunity. For instance, multifunctional natural microalgae and oncolytic viruses. Microalgae, a recently discovered photosensitizer, offers significant advantages and considerable development potential. Microalgae are abundant in natural photosynthetic pigments, including carotenoids and chlorophyll (Wang et al., 2025). These organisms have the capacity to directly produce ROS to induce tumor cell death in the presence of light with specific wavelengths (Zhou et al., 2023; Zhang et al., 2024; An et al., 2024). It shows that microalgae is a more cost-effective and accessible alternative to synthetic photosensitizers because microalgae are amenable to cultivation, requiring only basic nitrogen and carbon sources, which further enhances their economic viability. In the context of tumor treatment, microalgae exhibit a distinctive advantage in hypoxic tumor regions. For instance, chlorophyll has the capacity to spontaneously generate oxygen under light excitation, thereby alleviating tumor hypoxia and significantly enhancing the efficacy of PDT killing tumor cells. Furthermore, microalgae have also been demonstrated to exhibit favorable biocompatibility and immunomodulatory functions (Pan et al., 2013; Chan et al., 2009; Kuznetsova et al., 2017). The polysaccharides and other immunologically active compounds contained therein have been shown to enhance the activity of key immune cells such as macrophages, dendritic cells, and NK cells to activate anti-tumor immune responses and counteract the immunosuppressive phenomena in the tumor microenvironment. These comprehensive characteristics underscore the potential for the development of microalgae as a multifunctional, cost-effective, and biocompatible photosensitizer in the combined strategy of tumor photodynamic therapy and immunomodulation, which is a highly promising avenue for further research.

Oncolytic viruses represent a highly versatile platform for cancer therapy, exhibiting a dual mechanism of action: direct oncolytic activity and the activation of anti-tumor immune responses (Bommareddy et al., 2018; Xi et al., 2024; Ji et al., 2024). Through the application of genetic engineering techniques, these viruses can be modified to express photosensitizer proteins, thus providing a targeted alternative to conventional photosensitizers. This combined strategy enhances overall anti-tumor efficacy by improving tumor-selective toxicity and modulating the tumor microenvironment (TME). The combination of oncolysis, immunostimulation and localized photodynamic action has the potential to create a new avenue for the development of more precise, potent and systemic anti-tumor therapies. In Shimizu’s research, an oncolytic herpes simplex virus G47-KR that can express the photosensitising protein Killer RED (KR) was constructed (Shimizu et al., 2023). G47-KR has been demonstrated to exhibit both the cytotoxic effect of an oncolytic virus and the capacity to release ROS through KR, a process that facilitates tumor cell killing and augments the infiltration of immune cells within the immune microenvironment.

These next-generation PSs constitute a significant advance for PDT-based immunotherapy. Their design mitigates historical drawbacks of low ROS efficiency in tumor and biocompatibility, thereby sustaining potent antitumor effects. A key gap remains in understanding their precise mechanisms of immune activation, especially within the complex and dynamic tumor immune microenvironment where PDT elicits immunotherapy gradually. Thus, clarifying the immune mechanisms of these materials will pave the way for precisely modulated immunotherapy capable of reversing immunosuppression and elevating response efficacy.

## 3 Development prospects of PDT-induced tumor immunotherapy

PDT-induced immunotherapy represents a promising paradigm in oncology, with demonstrated antitumor efficacy. However, its clinical translation faces a significant gap due to the dual challenges of restricted light penetration and tumor hypoxia. Innovative approaches, including advanced PSs employing photoconversion strategies, are in preclinical development but await clinical confirmation. The core issue of light delivery depth persists, especially for treating deep or disseminated disease. Concurrently, the field lacks a rational framework for PS design and a detailed understanding of immune activation mechanisms. Current reliance on complex natural product-derived PSs highlights the need for strategies to develop synthetically accessible and stable agents. Ultimately, the most critical barrier to widespread adoption is the absence of standardized treatment parameters and validated efficacy assessment criteria for PDT immunotherapy. Addressing this deficiency is imperative for transforming this promising approach into a robust and reliable clinical modality. Furthermore, current studies have challenged the prevailing view that PDT-triggered apoptosis and ER stress are crucial for ICD, suggesting that the key mechanism underlying the induction of ICD still requires further investigation (Thibaudeau et al., 2025).

A significant challenge in optimizing PDT-induced immunotherapy is the TME, which is a dynamic, immunosuppressive niche that plays a critical role in determining treatment response (He et al., 2025). The TME and tumor cells exist in a state of co-evolution, engaging in constant crosstalk via extracellular vesicles, cytokines, and paracrine factors. This interplay is a driving force behind tumor progression by regulating processes such as vascular behavior, cell metabolism, and metastatic spread (Badve and Gökmen-Polar, 2023; Yin et al., 2023; Aliazis et al., 2025). Consequently, therapeutic strategies that target only the tumor cells may inadvertently exacerbate pro-tumorigenic pathways within the TME, ultimately limiting efficacy and potentially promoting recurrence and metastasis. The development of multifunctional PSs or combination regimens offers a promising path forward. The objective of such approaches is threefold: firstly, to eradicate tumor cells via ROS; secondly, to reverse immunosuppression; and thirdly, to promote immune cell activation and establish durable antitumor memory. For instance, flavonoids and microalgae-based agents, when used in conjunction with photosensitizers, have demonstrated potential in reversing immunosuppressive cues and stimulating immune activation, though further mechanistic investigation is required.

PDT serves as a pivotal link between innate and adaptive immunity, effectively converting immunologically “cold” tumors into inflamed, immunogenic “hot” tumors. This immunomodulatory effect forms the cornerstone of its synergy with established cancer treatments, particularly immunotherapy. Substantial preclinical and clinical evidence demonstrates that PDT enhances the efficacy of immune checkpoint inhibitors (e.g., anti-PD-1/PD-L1), promoting sustained antitumor immunity and abscopal responses. Representative studies include the use of a targeted photosensitizer (IR700DX-6T) with epigenetic and immune therapy to overcome immunosuppression (Zhou et al., 2024), and the application of PDT to sensitize checkpoint-inhibitor-resistant pancreatic tumors to anti-PD-1 treatment (McMorrow et al., 2025). These findings illuminate viable paths to overcome primary and adaptive resistance.

Despite this promise, the majority of PDT-based combination regimens remain exploratory, underscoring an urgent need to expedite clinical translation. A fundamental challenge resides in the intricacy and heterogeneity of treatment responses, which are driven by dynamic tumor microenvironmental factors. Advancing this field will require biomarker-guided strategies, including monitoring immune infiltration, molecular signatures and epigenetic modifications, in order to predict efficacy, manage toxicity and select patients for precision combination therapies (Darvin et al., 2018). Consequently, the path to effective clinical integration of PDT with immunotherapy hinges on our ability to navigate this biological variability, underscoring the need for personalized approaches.
